# Antitumor Effects of Combining Docetaxel (Taxotere) with the Antivascular Action of Ultrasound Stimulated Microbubbles

**DOI:** 10.1371/journal.pone.0052307

**Published:** 2012-12-20

**Authors:** David E. Goertz, Margarita Todorova, Omid Mortazavi, Vlad Agache, Branson Chen, Raffi Karshafian, Kullervo Hynynen

**Affiliations:** 1 Department of Medical Biophysics, University of Toronto, Toronto, Canada; 2 Sunnybrook Research Institute, Toronto, Canada; 3 Department of Physics, Ryerson University, Toronto, Canada; Northwestern University Feinberg School of Medicine, United States of America

## Abstract

Ultrasound stimulated microbubbles (USMB) are being investigated for their potential to promote the uptake of anticancer agents into tumor tissue by exploiting their ability to enhance microvascular permeability. At sufficiently high ultrasound transmit amplitudes it has also recently been shown that USMB treatments can, on their own, induce vascular damage, shutdown blood flow, and inhibit tumor growth. The objective of this study is to examine the antitumor effects of ‘antivascular’ USMB treatments in conjunction with chemotherapy, which differs from previous work which has sought to enhance drug uptake with USMBs by increasing vascular permeability. Conceptually this is a strategy similar to combining vascular disrupting agents with a chemotherapy, and we have selected the taxane docetaxel (Taxotere) for evaluating this approach as it has previously been shown to have potent antitumor effects when combined with small molecule vascular disrupting agents. Experiments were conducted on PC3 tumors implanted in athymic mice. USMB treatments were performed at a frequency of 1 MHz employing sequences of 50 ms bursts (0.00024 duty cycle) at 1.65 MPa. USMB treatments were administered on a weekly basis for 4 weeks with docetaxel (DTX) being given intravenously at a dose level of 5 mg/kg. The USMB treatments, either alone or in combination with DTX, induced an acute reduction in tumor perfusion which was accompanied at the 24 hour point by significantly enhanced necrosis and apoptosis. Longitudinal experiments showed a modest prolongation in survival but no significant growth inhibition occurred in DTX–only and USMB-only treatment groups relative to control tumors. The combined USMB-DTX treatment group produced tumor shrinkage in weeks 4–6, and significant growth inhibition and survival prolongation relative to the control (p<0.001), USMB-only (p<0.01) and DTX-only treatment groups (p<0.01). These results suggest the potential of enhancing the antitumor activity of docetaxel by combining it with antivascular USMB effects.

## Introduction

Microbubbles (MBs) are systemically injected encapsulated micron sized (1–10 microns) bubbles that oscillate in response to incident ultrasound and are currently in clinical use as diagnostic contrast agents. When MBs are employed under therapeutic ultrasound exposure levels, it is well established that their oscillations are capable of increasing the permeability of microvessels and thereby enhancing the extravasation of molecules, nanoparticles and therapeutic agents [1], [2]. These effects have been explored in a range of tissue types and the results to date suggest the considerable potential of this approach as a means of augmenting the local delivery of therapeutic agents in the treatment of a range of diseases. Work to promote the delivery of anticancer agents into tumor tissue through microbubble potentiated microvascular permeability enhancement is also being undertaken [3]. This has been motivated by the recognition that a major barrier to the effectiveness of many anticancer agents is that they are unable to reach therapeutic concentrations within tumor tissue [4], [5]. In brain tumors, results have begun to emerge showing improved therapeutic effects by employing ultrasound stimulated microbubbles (USMB) with liposomal drug formulations under conditions that produce enhanced drug extravasation [6]–[8]. Outside the brain, results have recently been obtained indicating that the use of USMBs with anticancer agents can result in improved delivery and therapeutic effects relative to the drug only case [9]–[12]. With the exception of brain tumors, where the blood-brain-barrier presents a significant drug delivery obstacle, it must also be considered that in many circumstances the penetration of antitumor agents into tumor tissue is not inhibited by their inability to extravasate from the bloodstream. Rather, issues such as elevated interstitial fluid pressure levels and their uptake and sequestration in perivascular regions will limit the transport and distribution of anticancer agents within tumor tissue [13]. It is therefore of interest to consider possible alternative approaches for employing USMBs in conjunction with anticancer agents that do not rely simply upon the enhancement of extravasation from the vascular compartment.

In addition to permeabilization effects, which can be achieved at relatively low ultrasound (US) amplitudes, the stimulation of MBs with sufficiently high US amplitudes can induce microvascular damage [14]–[16]. While the precise mechanisms of damage are not well understood, there is evidence that this process can result in hemorrhage, edema, and thrombus formation [14], [15]. These effects have been linked to violent MB oscillations and collapse (inertial cavitation) [14], [15], which can create high local temperatures, elevated vascular wall shear stresses, directional ‘jets’ and the production of free radicals [17], [18]. The antivascular effects of US stimulated MB treatments (USMB) in tumors have also begun to be investigated, with initial work showing that they can cause an acute shutdown of blood flow [19]–[23]. In recent work, it was demonstrated that these effects can occur rapidly (<1 minute) and preferentially within tumor centers [23], and that tumor growth inhibition can be achieved with this approach [21], [22], [24].

While these results suggest that the antivascular action of USMBs may have potential as a monotherapy, it is also of interest to investigate how this approach can be exploited in conjunction with anticancer agents. To this end, it is useful to consider the substantial body of research that has accumulated over the past two decades examining the antitumor effects of small molecule vascular disrupting agents (VDAs) [25], [26]. Indeed, the rapid shutdown of blood flow within tumors following USMB treatments appears to have significant parallels to the effects of vascular disrupting agents [25], [27]. VDAs act upon the established but fragile and aberrant tumor vasculature, and can induce widespread necrosis within tumor centers. As a monotherapy, VDAs have however exhibited only limited effectiveness in achieving sustained antitumor effects [27], [28]. This has been attributed in part to a ‘vascular rebound effect’, whereby the rims of tumors are less affected by the treatment, and can act as a site for revascularization and re-growth. VDAs have therefore been investigated in combination with other therapeutic approaches [29] such as radiotherapy, antiangiogenic therapy, or chemotherapy [30]–[32].

The focus of the present paper is to investigate the combination of antivascular USMB treatments with a chemotherapeutic agent. The biological rationale for combining VDAs with chemotherapy is based on several key tenets [27], [33], [34]. The factor that is generally considered to be the most significant is that VDAs and chemotherapy will act in a complementary manner on different tumor compartments. VDAs preferentially damage the fragile tumor neovasculature and thereby induce necrosis in the tumor centers, whereas cytotoxic chemotherapeutic agents preferentially affect highly proliferating well perfused tumor rims. A secondary rationale is that if a chemotherapeutic agent is circulating at the time of VDA administration, the ensuing vascular shut down may act to entrap agent within tumor tissue. It is recognized that there can be other contributing factors such as possible biochemical interactions between the drugs on the same tumor compartment and the impact of VDA treatment on modifying the tumor microenvironment, though these effects can also potentially act to hinder complementary action [34].

Numerous preclinical studies and a substantial amount of early clinical work has been carried out combining VDAs with a wide range of chemotherapeutic agents [25], [27], [35], [36]. In general, it has been found that the addition of chemotherapy enhances to some extent the antineoplastic effects of VDAs. The degree of therapeutic enhancement is highly variable, ranging from being negligible to supra-additive. Taxanes (paclitaxel and docetaxel) in particular have been shown to exhibit significant, beyond additive, antitumor effects when combined with VDAs. This has been demonstrated in a range of preclinical tumor models and is also undergoing initial clinical evaluation [27], [37]–[39].

In this study we examine the combination of antivascular USMB treatments with docetaxel (Taxotere). Docetaxel (DTX) is widely employed in the treatment of a broad range of solid tumor types including prostate, breast and head and neck cancers [40]. The work is carried out in a preclinical prostate carcinoma model (PC3) implanted in nude mice. The acute effects of treatments on tumor perfusion, apoptosis, and necrosis are examined. Longitudinal experiments are carried out to assess the impact of treatments on tumor growth and animal survival.

## Materials and Methods

### Mice and Tumors

Human prostate carcinoma PC3 cells were purchased from the American Type Culture Collection (Manassas, VA). Cells were maintained in RPMI medium supplemented with 10% fetal bovine serum (FBS), penicillin (100 U/mL) and streptomycin (100 µg/mL) and were cultured at 37°C in a 5% CO_2_/95% air atmosphere. Cells were maintained at exponential growth and passaged twice weekly for *in vivo* studies. PC3 tumors were established by the subcutaneous (s.c.) injection of 50 µL of 1×10^6^ cells/mL in the right hind leg of 4–6 week-old male athymic nude mice (NIH Swiss Nude from Taconic, Germantown, NY, US). Mice were housed five per cage with sterile food and water given *ad libitum*. Mice reaching study endpoints or ethical endpoints were sacrificed by cervical dislocation while anesthetized under 1–3% isoflurane. Ethical endpoints included reaching a maximum allowable tumor size (institutionally limited to 17 mm in diameter) or signs of distress. All animal procedures were approved by the Sunnybrook Research Institute Animal Care and use Committee and conformed to the guidelines set out by the Canadian Council on Animal Care.

### Drug and Microbubbles

Docetaxel (Taxotere, Aventis Pharmaceuticals, Inc.) was obtained from the hospital pharmacy at concentration of 40 mg/ml. The drug was diluted in an ethanol-saline mixture to a final 1 mg/ml concentration and DTX treated mice received a dosing level of 5 mg/kg. The MBs employed in this study were an experimental agent obtained from Artenga Inc. (Ottawa, Canada) which were octofluropropane gas cores encapsulated with shells comprised of sorbitan monostearate (Span 60) and polysorbate 80 (Tween 80). These MBs have a peak in their volume distribution at 3.8 microns and a number weighted mean diameter of 2.13 microns when measured with a Coulter counter in a size range of 1–12 microns. The MBs were activated immediately before use and diluted in saline to a final concentration of 30% (v/v) for injection. The injections were at a dose level of 2.1·10^5^ bubbles/g of mouse weight. A direct comparison of this dose level with other MB agents is difficult due to, for example, differences in size distributions. As a point of reference, if the number density of bubbles in the 1–12 micron range for the commercial agent Definity™ were considered [41] this would correspond to a 1 µl injection into a 25 g mouse, or a 40 µl/kg dosing level.

### Therapeutic Ultrasound Configuration

**Figure 1:**
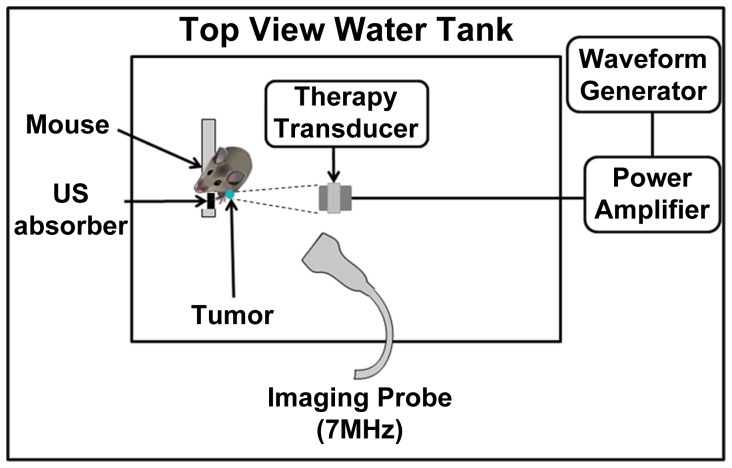
Overview of the apparatus employed to expose and image mice. Anesthetized mice are situated vertically in a water tank (35°C) for acoustic coupling with the ultrasound transducers. A therapy transducer is focused upon the tumor and controlled pulses are generated and transmitted. An US imaging transducer is used to image MBs entering into the tumor.

An overview of the experimental configuration is shown in Fig. 1. Animals were anesthetized with s.c. injections of 0.1 ml/10 g of 4∶1 ratio ketamine-HCl (100 mg/ml) and xylazine-HCl (20 mg/ml), catheterized as described below, and mounted vertically in a 35°C water tank immersed to the shoulder region, the general approach of which has been previously reported [42]. Therapeutic US exposures were applied with a spherically focused 1 MHz transducer (Valpy Fisher, Hopkinton MA; diameter 3.75 cm; focal length 15 cm; −6 dB beam width at focus of 1.05 cm) with its beam oriented approximately normal to the upper tumor surface. A single transducer position was employed with the focus located at the center of the tumors, which had lateral dimensions that were always within the 6 dB beamwidth of the transducer. A previously reported sonication scheme was employed, comprised of bursts of 50 0.1 ms long pulses spaced 1 ms apart, which were repeated at 20s intervals for a duration of 3 minutes. The relatively short individual pulse lengths were used to reduce the potential for standing wave effects, in consideration of the relatively weakly focused transducer beam. The timing between bursts was based on pilot work examining contrast reperfusion time [23]. Peak negative pressures at focus were 1.65 MPa, as measured with a calibrated 0.2 mm needle hydrophone (Onda, Sunnyvale, CA).

### Treatment Procedure

There were 4 mouse groups in this study: control (sham treatment), DTX-only, USMB-only and a combined USMB+DTX treatment group. An overview of the experiments conducted is provided in a later section. All mice underwent identical procedures with respect to anesthetization, catheterization, time in water tank (∼30 minutes), and the injection of MBs. In the case of control and DTX-only groups, the mice received MB injections but the US exposure was not performed. All drug and MB injections occurred through an indwelling 26 Ga tail vein catheter. For the combination USMB+DTX group, the diluted drug was injected 10 minutes prior to USMB treatments being initiated. This has been shown to be approximately equal to the plasma half-life of docetaxel in mice [43] and enabled consistent timing relative to USMB treatments. For the USMB and USMB+DTX groups, the US pulsing sequence commenced 15 s after the intravenous injection of a 50 µl injection of diluted MBs. This process was repeated at 10 minute intervals for a total of three injections.

### Ultrasound Flow Monitoring

In addition to MBs being employed for therapeutic purposes in this study, their influx into tumors was imaged to gain insight into the effects of treatment on blood flow, and to provide an indication of MB destruction following individual therapeutic US bursts. US imaging of MBs was performed with a Toshiba Aplio (Otawara, Japan) using a 7 MHz probe operating in contrast imaging mode at a low mechanical index (0.05) and frame rate of 11 Hz. The probe was situated outside the therapy beam, with its imaging plane intersecting at an angle of 60 degrees. Imaging data was collected from the time of bolus injection, which permitted monitoring of MB concentration during exposure to enable a qualitative assessment of short-term effects of the treatment on tumor perfusion. The resulting data was then processed in the following manner. First, to extract information relevant to blood flow, the Aplio analysis software was employed to select two regions of interest (ROIs) within each tumor: a central (1/2 distance from center to edge) and a peripheral (outer 1/2) region. This resulted in two contrast intensity curves (related to MB concentration as a function of time following bolus injection). These curves were then analyzed to estimate the slope of the initial rise (10–90%) during the bolus influx, a procedure that has been previously employed to extract information related to blood flow in mice [44]. This analysis was performed for all injections. The ratio of the 3^rd^/1^st^ bolus slope measurements was then calculated, which was used a metric of the extent to which the therapy affected blood flow in the tumor. These values were then normalized with respect to those for the control tumors.

### Ultrasound Cavitation Detection

For a subset of mice (n = 2), an experiment was conducted to record the acoustic signals emitted from oscillating MBs (‘cavitation’ signals) within tumors during exposure to the therapeutic US bursts. This was accomplished by replacing the imaging transducer with a single element focused transducer (750 kHz; focal length 7.5 cm, diameter 2.5 cm; Valpey Fisher, Hopkinton MA). The focus of this transducer was localized to the tumor, overlapping with the therapy transducer focus. Signals from this transducer were digitized (Alazartech, Montreal, Canada) during the therapy US pulses. The frequency content (average power spectra) of the signals was then calculated with custom written Matlab code (Natick, MA).

### Tumor Size Measurements

Tumor growth was monitored weekly using 3D high frequency US (Vevo770 US system, VisualSonics, Toronto, Canada) [45]. Volumetric ultrasound images were acquired with a 20 MHz or 30 MHz transducer probe, depending upon tumor size. Tumor volumes were then measured off-line from the 3D images using Vevo 770 v3.0 software. For the growth study *(Experiment Set 2*), volume measurements were conducted once per week for the duration of the experiments.

### Overview of Experimental Groups

Two types of experiments were conducted: acute and longitudinal.

*In Experimental Set 1*, experiments were conducted on tumors in the range of 120–150 mm^3^ for the purposes of assessing the acute response to a single treatment session. At the 24 hour point, tumors were excised to examine tumor morphology by Haematoxylin and Eosin staining (H&E), apoptosis, and DiOC_7_ perfusion analysis (see Histology methods section). There were 4 groups (n = 5–7 per group) in this experiment: control (sham treatment with MB only injection), DTX-only, USMBs only, and combined USMB+DTX treatment. In all cases, mice were catheterized and were situated in the water tank for a period of approximately 40 minutes. An additional set of mice (n = 2) underwent treatment and were sacrificed for H&E histology within 1 hour of the treatment. During their treatment cavitation signals were recorded as described above.

*In Experiment Set 2* the impact of treatments on tumor growth and survival was assessed. Mice were randomly assigned to one of four experimental groups when tumor volumes reached 60 to 80 mm^3^ with a larger number of mice entering each group (shown in Table 1). The groups were control (MBs only- sham treatment), USMBs, once a week intravenous injection of DTX and MB, and combined USMBs and DTX treatment. The treatment procedures were performed on 4 consecutive weeks (days 0, 7, 14 and 21). Animals were sacrificed when they reached institutional ethical endpoints associated with tumor burden. We note that a subset of animals were not able to be re-catheterized in successive weeks, in which case they were included in the tumor growth data set until that point, and excluded thereafter. The animal numbers per group available for growth measurements in weeks 0–3 were: control (n = 6–7), USMB (n = 6–8), DTX (n = 6–8) and USMB+DTX (n = 6–10), where the range in numbers for a given group reflects the difference between the number entering week 0 (higher number) and those completing the full 4 weeks of treatment (lower number). Only animals successfully undergoing procedures for the full 4 weeks were considered in the survival curves. We note that an US-only group (i.e. no MBs) was not included in the study as the exposure levels are well below what would be expected to induce bioeffects in the absence of injected MBs. Similarly, MBs in the absence of US are not expected to affect tumor growth and therefore it was not considered necessary to have a saline only group. At week 0 US flow processing was performed to examine flow changes occurring during a single treatment as described in the methods section.

**Table 1:** Summary of growth curve statistics.

	Groups					
Week	Control vs DTX	Control vs USMB	Control vs USMB+DTX	USMB vs DTX	USMB+DTX vs USMB	USMB+DTX vs DTX
**1**	–	–	–	–	–	–
**2**	–	0.055 (*ns)	0.012 (*ns)	–	–	0.034 (*ns)
**3**	–	0.084 (*ns)	0.0008 (*p<0.01)	–	0.0008 (*p<0.01)	0.012 (*ns)
**4**	n/a	n/a	n/a	–	0.0016 (*p<0.01)	0.002 (*p<0.01)
**5**	n/a	n/a	n/a	n/a	0.00072 (*p<0.01)	n/a

Tabulation of p-values for tumor volume differences between groups in the growth experiments. The unbracketed p-values are derived from an unpaired two-tailed Student’s t-test, and the bracketed values indicate the level of significance with a *post hoc* Bonferroni test (denoted *p). ‘-‘ indicates p-value was >0.1 for the Student t-test and ‘*ns’ within brackets indicates that the *post hoc* Bonferroni test had a p-value of >0.05. n/a indicates that at least one group was not present in the growth curves at that point (i.e. drop-outs occurred due to tumor burden end-points). By week 3 the combined USMB+DTX group had a significantly (*p<0.01) lower mean tumor volume than the control group and at week 4 and after was also significantly (*p<0.01) lower than the USMB and DTX groups.

A total of 54 mice were used in this study. The group sizes were within the range that has previously been employed in numerous other studies to assess the combined effect of two treatment approaches (i.e. chemotherapy and antivascular agents) and the numbers were sufficiently large to result in statistically significant differences between groups.

### Histology

A histological evaluation of excised tumor tissue was conducted for mice involved in *Experiment Set 1* at the 24 hour time point following treatment. DiOC_7_ injection was performed immediately prior to sacrifice and tumor tissue was harvested and frozen at −80°C in optimum cutting temperature medium (OCT, SAKURA Finetek USA Torrance, CA). Two adjacent 5 µm cryosections were then taken through the central region of tumors, with one undergoing H&E staining and analysis and the second being analyzed first for DiOC_7_ and then terminal deoxyribonucleotidyltransferase (TdT)-mediated dUTP nick end-labeling (TUNEL) staining for apoptosis analysis.

*H&E procedures*: Tumor sections were processed for H&E staining, the images were acquired with a Mirax Scanner (Carl Zeiss Inc., Germany) using a 20× objective and subsequently analyzed for the percentage area of necrosis.

*DiOC_7_ perfusion staining:* Microvascular perfusion was assessed using DiOC_7_ perfusion staining. DiOC_7_ was purchased from Molecular Probes (Invitrogen) and reconstituted in 75% (v/v) DMSO:25% PBS. Dye was injected intravenously (i.v). through the tail vein in the form of a 100 µL bolus at a concentration of 0.5 mg/mL. Injections were performed while the mice were anesthetized with 1–3% isoflurane. The tissue sections were digitized within 24 hours with an Olympus fluorescent microscope (Model BX50) at 10× with an excitation wavelength of 488 nm and an emission filter for 515 nm. The resulting images were then analyzed for the density of perfused vessels [46] with custom written Matlab (Natick, MA) code.

*Apoptosis:* The degree of apoptosis was assessed by immunohistological examination of DNA fragmentation using TUNEL reaction on the previously scanned DiOC_7_ slides. Diamidino-2-phenylindole (DAPI, Sigma) counterstain was used to mark all the nuclei and to quantify the percentage of apoptosis by dividing the TUNEL-positive cells over the total DAPI-stained nuclei [47]. Tumor sections were fixed with ice-cold acetone for 5 min, rinsed in PBS prior staining and incubated with TdT in the presence of fluorescein isothiocyanate (FITC)-coupled dUTP (Promega, Madison, WI, USA, 1∶200 dilution). DAPI was added at 1 µg/mL for 5 minutes at the final step and slides mounted with a cover slip. Fluorescence was assessed on an Olympus fluorescent microscope as described above by imaging FITC labeled cells (excitation 490 nm and emission 525 nm) and DAPI (excitation 360 nm, emission 450 nm) counter stained nuclei.

### Statistical Analysis

A statistical comparison of tumor sizes, DiOC_7_ perfused vessel counts, necrotic area and apoptotic cell counts between groups were performed using a two-tailed paired Student's *t*-test. In addition to presenting these p-value calculations, the results of a *post hoc* Bonferroni analysis are also reported for the comparison of multiple pairs of groups^48^. The significance level for the family of comparisons was set at 0.05. For the histology and the growth data up to the 3 week point there are four treatment groups present (including sham/control) with six possible paired comparisons, for which pairwise p-values less than the Bonferroni corrected threshold of 0.05/ = 0.0083 were considered to be statistically significant. These p-value levels of significance are denoted ‘*p’ to distinguish them from the Student’s t-test results. For the growth data beyond 3 weeks the intact groups were reduced such that at 4 weeks there were three possible paired comparisons (0.05/3 = 0.0167) and at 5 weeks there was a single pair (0.05/1 = 0.05).

## Results

### Flow Effects

**Figure 2:**
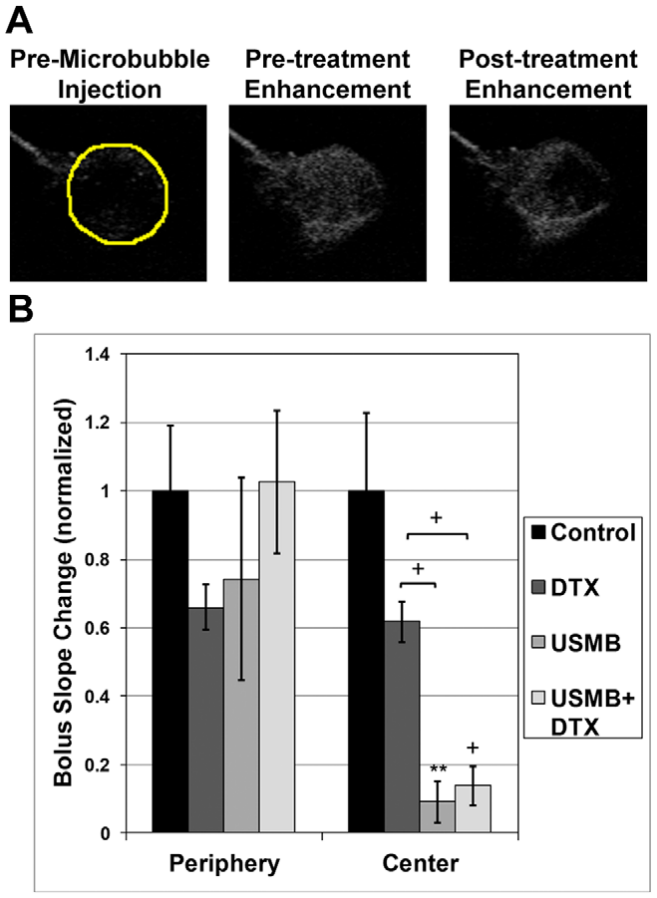
USMB treatments reduce blood flow in tumors. a) Example US contrast images at peak enhancement following MB injection prior to treatment (left) and after two therapy injections (right) illustrate qualitatively the reduction of perfusion resulting from the treatment, which preferentially affects the central regions of the tumor. b) A quantification of the results (based on slope processing) indicates significant flow reduction in both the central and peripheral regions, with the central regions being more substantially affected by the treatment. Mean and SEM values shown, and **, + represent p<0.01 and p<0.001 respectively for the Student’s t-test analysis. The *post hoc* Bonferroni analysis showed significance between the USMB and both the control and DTX groups (*p<0.05), and between the combined USMB+DTX and both the control and DTX groups (*p<0.01).

Fig. 2A shows example images of the peak contrast enhancement in a Week 0 tumor for the first and third MB injections. In Fig. 2A (middle) it can be seen that the tumor is well perfused with MBs on the first injection, prior to receiving any USMB treatment. For the third injection (Fig. 2A, right), after the tumor has undergone two of the three treatment sequences, a perfusion deficit can be observed within its center. A quantification of the contrast imaging flow processing results for control and USMB tumors at Week 0 is shown in Fig. 2B. After 2/3 of a treatment session, there was a significant reduction of flow within the central regions of the tumors for the USMB group versus both the control (11.2 fold reduction; *p<0.05) and DTX (9.1 fold; *p<0.05) only groups. For the USMB+DTX group there was also a significant flow reduction relative to the control (12 fold; *p<0.01) and DTX only (9.1 fold; *p<0.01) groups. This trend is also observed in the peripheral regions, though the effect is less pronounced. We note that for subsequent treatment weeks it was observed that there was a degree of flow re-establishment within the central tumor regions for the USMB and USMB+DTX groups. A quantitative analysis of the US based flow measurements in later weeks is not presented since with tumor growth there is the gradual development of poorly perfused necrotic regions within the tumors and as mean tumor sizes vary between groups it is not possible to separate these size related effects from potential differences associated with treatments.

**Figure 3:**
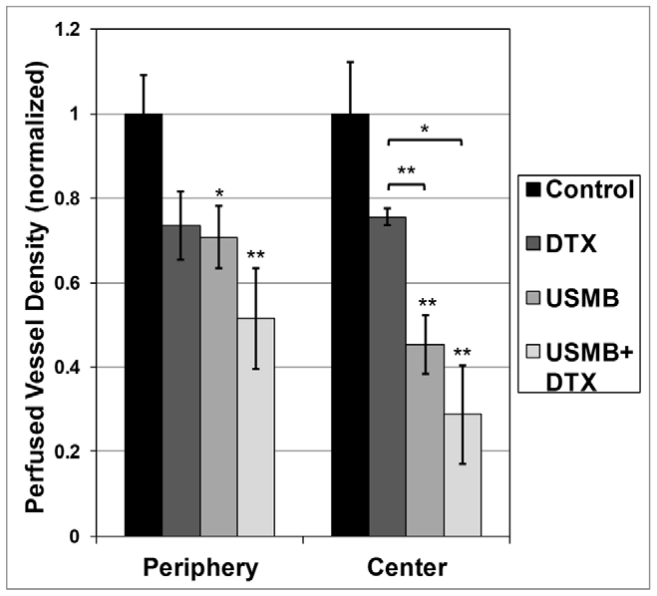
DiOC_7_ staining shows USMB treatments reduce tumor perfusion. A quantitative analysis of the DiOC_7_ results indicates that there has been a significant reduction in the number of perfused vessels in combined DTX +USMB-treated tumors (24 hour point) relative to single treatments. Mean and SEM values shown, where * and ** represent p<0.05 and 0.01 respectively for the Student’s t-test analysis. For the tumor centers, the *post hoc* Bonferroni analysis was significant (*p<0.05) between the USMB and both the control and DTX groups, and between the combined USMB+DTX and both the control and DTX groups. Significance was not reached with this analysis for the peripheral regions.

The quantified 24 hour DiOC_7_ perfusion staining results are shown in Figs. 3A and B for the central and peripheral regions respectively. In both regions, tumor perfusion is lower for the USMB and combined USMB+DTX treatment groups relative to the control group. For the central region, this reduction in perfusion is more pronounced, and was significantly lower in the USMB compared to both the control (2.2 fold reduction; p<0.05) and DTX (1.6 fold; *p<0.05) groups and between the combined USMB+DTX and both the control (3.5 fold; *p<0.01) and DTX (2.6 fold; *p<0.02) groups.

**Figure 4:**
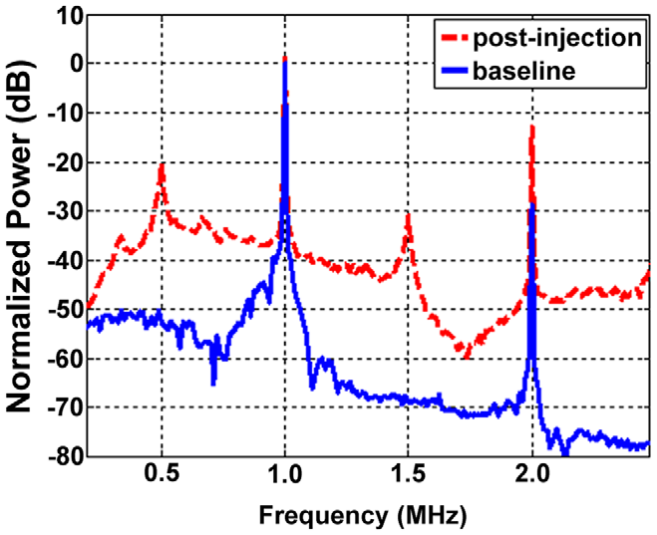
Example ‘cavitation’ signals recorded from the MBs within the tumor during exposure to therapy pulses. The ‘baseline’ signal (blue solid) is taken prior to MB injection and arises from scattering of the incident US by tissue. Here a clear 1 MHz component (transmit frequency) along with a 2 MHz signal associated with (nonlinear) propagation of the US pulse. Signals outside these frequencies are associated primarily with noise. For the MB signals (red dashed) there are also pronounced peaks at 0.5 and 1.5 MHz, referred to as subharmonic (subH) and ultraharmonic (UH) signals which occur in the presence of strongly oscillating MBs. The remaining substantial energy present across a wide range of frequencies is a hallmark of ‘inertial’ cavitation, indicating the violent oscillations of MBs during the therapy pulses.

During exposure pulses, which induce MB destruction, the frequency content of the cavitation signals emitted by the MBs are shown in Fig. 4. The presence of signals at frequencies outside 1 MHz (the transmit frequency) are linked to MB oscillations. The presence of peaks at 0.5 (subharmonic) and 1.5 MHz (ultraharmonic) are indicators of substantial MB oscillations, and the broadband signal across a range of frequencies is associated with ‘inertial cavitation’, which is a hallmark of violent MB oscillations.

### Tissue Histology

**Figure 5:**
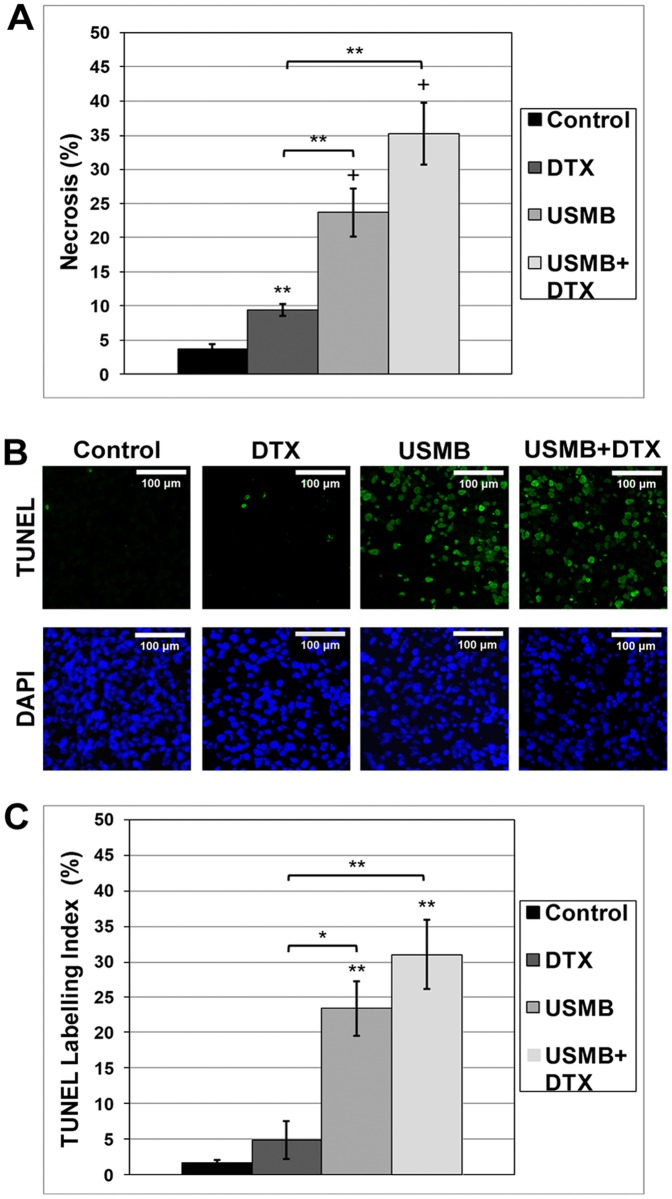
Quantitative histologic analyses of tumors at the 24 hour point. a) A quantification of H&E stained tumors shows a higher percentage area of necrosis for all treated tumor groups (Bonferroni analysis *p<0.05) relative to control tumors and the USMB+DTX group is also significantly lower (*p<0.05) than the USMB group. b) Example images for control (left) and 24 hr post-USMB treatment (right) tumors stained for TUNEL (green, top) and DAPI (blue, bottom) and H&E stained tumors indicate a higher level of apoptosis is observed in the treated relative to control tumors. c) A quantification of these results showed that both the USMB and combined USMB+DTX groups had significantly higher apoptosis levels than the control group (Bonferroni analysis *p<0.01) and the USMB+DTX group was significantly lower than the DTX only group (*p<0.02). Mean and SEM are plotted *, ** and + indicate Student’s t-test p-values of less than 0.05, 0.01 and 0.001 respectively for differences between means of groups.

The H&E necrosis analysis for the acute experiments is shown in Fig. 5A. A quantification of the results showed significantly higher percentage areas of necrosis for the DTX (2.4 fold increase; *p<0.05), USMB (5.9 fold; *p<0.001) and USMB+DTX (9.7 fold; p<0.001) groups relative to the control tumors. Further, the USMB+DTX group also had significantly more necrosis (1.6 fold; *p<0.05) than the USMB group.

Example TUNEL and DAPI staining results are shown in Fig. 5B, and a quantification of the results for apoptosis at the 24 hour point are in Fig. 5C. There were significantly higher levels of apoptosis for both the USMB and combined USMB+DTX groups compared to the control group (*p<0.01) and the USMB+DTX group was significantly higher than the DTX only group (5.0 fold; *p<0.02). For both the necrosis and apoptosis results, the levels were higher for the USMB+DTX than for the USMB group, though this did not reach statistical significance.

### Growth Experiments

**Figure 6:**
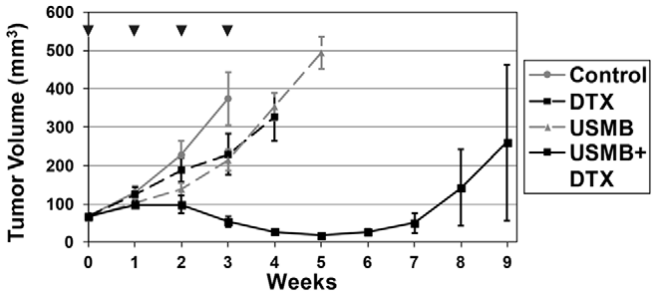
Tumor growth as a function of time. The results show that the USMB- and DCTX-only treatments have a mild tumor growth inhibitory effect relative to the control group. The combined USMB+DCTX group produces significant growth inhibition relative to the individual treatment groups after a week 1. Significant (p<0.01) tumor shrinkage for the combined USMB+DTX groups is observed in weeks 4–6. Note that the increase in volume with time for the USMB+DTX group in weeks 8 and 9 is largely associated with one individual tumor; without the inclusion of this tumor, the mean sizes at the 8 and 9 week points are 44.3 and 63.1 mm^3^ respectively. p-values for differences between group mean sizes are shown in Table 1. ▾ indicates the occurrence of a treatment.

**Figure 7:**
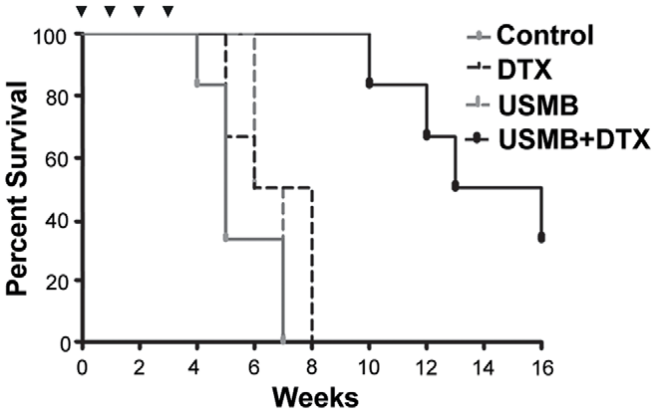
Survival curves for the growth experiments. Modest survival increases are associated with individual DTX and treatment USMB groups relative to control tumors. The combined USMB+DTX group produces prolonged survival relative to the control and both individual treatment groups. In particular, by week 8 the control and individual treatment groups have no surviving members, while the combined treatment group has 100% survival until week 9 and 50% survival at week 15. ▾ indicates the occurrence of a treatment.

The results for the longitudinal experiment are shown in Fig. 6 for growth and Fig. 7 for survival. A summary of the significance levels for size differences between groups as a function of time is shown in Table 1. Control tumors grew rapidly and began to reach endpoints due to tumor volume after week 3. The USMB and DTX individual treatment groups were not found to have produced a significant reduction in tumor size as a function of time, but did result in modest survival prolongation. The combined USMB+DTX treatment group tumors were significantly smaller than the control group at all weeks >2, (Bonferroni analysis *p<0.01) and from week 3 onwards with respect to the USMB group (*p<0.01) and in week 4 with respect to the DTX group (*p<0.01) (see Table 1 for summary). For the combined USMB+DTX treatment group, the tumors were significantly (*p<0.05) reduced in size for weeks 4–6 (e.g. 3.9 fold at week 5), relative to the week 0 starting point. Survival was also prolonged in the combined group relative to all other groups (Fig. 7). Tumor growth delays (TGD) were determined by the difference in time taken for the mean tumor volume to double and triple relative to the week 0 values in the treatment versus control animal groups. The TGD for doubling associated with the DTX, USMB and combined USMB+DTX groups were 0.1, 0.65 and 6.9 weeks respectively. The TGD for tripling associated with the DTX, USMB and combined USMB+DTX groups were 0.6, 1.0 and 6.8 weeks respectively. There was no significant reduction of the average body weights for any group over the course of the experiments (data not shown).

## Discussion

This study has demonstrated a pronounced enhancement of the antitumor activity of docetaxel through its combination with USMB treatments. The combined effects were significantly higher than both the individual treatment groups and were achieved under conditions where the USMBs induced antivascular effects.

The USMB group results obtained in this study are broadly consistent with other recent reports using relatively low (lower than employed in ablative high intensity focused ultrasound) US exposure levels to achieve antivascular effects in tumors. Both the US flow imaging and DiOC_7_ results demonstrate that a substantial vascular shutdown occurred in response to USMB treatments. Such a shut-down of blood flow has been reported in earlier work, and has been linked to increases in necrosis and apoptosis [21]. The induction of growth inhibition in tumors with USMB treatments at lower US intensities has also been observed [21], [22], [24]. While the US exposure levels employed in these studies is lower than would be used in ablative high intensity focused ultrasound (HIFU), it is notable that the experimental conditions do differ between these studies. Some have employed high duty cycle (fractional ‘on-time’ up to 0.5) exposures that produced macroscopic temperature elevations [19], [20] which themselves can induce bioeffects [48]. Others [21], including the present study, have found antivascular effects to occur at relatively low duty cycle levels that are not expected to be associated with substantial bulk tissue temperature elevations. Rather, due to the clear presence of vascular shut-down, it is interpreted that the growth delays are the result of flow shutdown effects. We note that the cavitation results shown in Fig. 4 suggest that, for the conditions employed in this study, inertial cavitation is occurring. This is consistent with the production of violent bubble collapse that can produce localized regions of high shear stress and temperatures, ‘jets’ [18], and an overexpansion or invagination of microvessels [49], which may act to induce vascular damage.

The central result of this study is that antivascular USMB treatments enhance the antitumor effects of docetaxel. A substantial amount of work related to the use of USMB treatments in combination with antitumor agents has been conducted previously, which has largely focused on promoting uptake though enhancing permeability. A number of studies have been conducted *in vitro*, generally using suspensions of tumor cells and microbubbles, which have demonstrated the improved intracellular uptake of anticancer agents and in some cases subsequently enhanced cell death [11], [50], [51]. However, for systemically injected contrast agents, MBs remain within the vasculature due to their sizes (∼1–10 microns) and will therefore not be expected to be in contact with tumor cells. The most relevant *in vivo* analogue of this *in vitro* approach requires the direct injection of MBs into tumor tissue. With this approach, it has been reported that combined USMB-drug treatments can result in growth inhibition, though significantly enhanced antitumor effects relative to drug only or USMB-only groups were not found [11], [12]. Studies using systemically (intravenously) injected MBs, the current clinically approved method for introducing agents into the body, have also been conducted with rationale of enhancing drug delivery into tumor tissue by increasing extravasation. Work in preclinical brain tumors has shown enhanced therapeutic effects under conditions where USMBs promote drug extravasation [6]–[8] by transiently disrupting the blood-brain barrier. For tumors situated outside the brain, a number of reports have demonstrated that USMB exposures can enhance the uptake of dyes, drugs and nanoparticles within tumors [1], [51]–[54]. Sorace et al [51] reported that USMBs enhanced paclitaxel uptake but the increase was not associated with improved antitumor activity relative to the drug only case. Using similar exposure conditions, albeit with high MB concentrations, Heath et al [9] found that combining USMB with cisplatin increased growth inhibition relative to the drug only case. The potential for USMB induce flow inhibition was not monitored in this study, though it was noted that there was no histologic evidence of USMB induced tissue damage or increases in apoptosis and the enhancements effects were therefore attributed to vascular permeability increases. In another recent study, the combination of vascular damaging USMB treatments with Doxil, a liposomal formulation of doxorubicin, was assessed [10]. These experiments were carried out under conditions that were shown to have histologic evidence of vessel damage resulting from USMB exposures, and for the combined drug and USMB group a reduction in tumor perfusion was measured. This work was motivated by improving drug uptake, as previous work had shown that USMB induced vascular damage could promote the uptake of 50–180 nm particles [53], and Doxil can be limited by issues of extravasation. The combination of USMB and drug treatment was found to produce growth inhibition relative to the drug group, but it was not reported that there was a statistically significant difference between the combined treatment group and USMB-only treatment, which is necessary to determine if the action of the drug was potentiated by USMBs. Therefore, while USMB treatments are promising as an approach to promote the uptake of anticancer agents into tumors, this work is at an early stage with respect to determining its potential to enhance the antineoplastic effects of anticancer agents.

While the conditions employed in the present study do not preclude permeability enhancement effects being a factor in achieving the results, they have functionally damaged the tumor vasculature as evidenced by the presence of sustained flow reductions. It is therefore useful to consider these findings in light of previous work conducted with VDAs and taxanes. Pronounced enhancements of the antitumor effects of taxanes have been achieved when combined with either of the two major classes of VDAs: tubulin binding agents and flavonoid derivatives. Tubulin binding agents selectively destabilize the cytoskeleton of proliferating endothelial cells (EC). This leads to endothelial cell rounding, and a cascade of ensuing events such as the exposure of basement membranes, transiently enhanced permeability, RBC extravasation, and a pronounced shut-down of blood flow [25], [27]. Macroscopically, tumor necrosis follows the administration of these agents. When these agents are combined with the taxanes paclitaxel or docetaxel, there is a supra-additive effect for inducing growth delays in preclinical tumors [55], [56]. Similarly, with the flavonoid derivative VDA DMXAA (a.k.a. vadimezan and AS404), highly synergistic (beyond additive) effects have been achieved with both paclitaxel and docetaxel for a number of tumor types [33], [37], [39], [57]. DMXAA has a tubulin independent mechanism of action involving biphasic direct and indirect antivascular effects. The biochemical pathways of DMXAA action are not fully understood, but EC apoptosis is known to be induced within 15–30 minutes [58], [59], a consequence of which is EC morphology changes, the exposure of microvascular basement membranes and platelet accumulation. Indirect antivascular and antitumor activity also occurs, in large part associated with the influx of neutrophils and the upregulation of a range of cytokines, such as tumor necrosis factor.

While the mechanisms responsible for the combined action of VDAs and taxanes are not fully understood, it is generally considered that the primary factor involved is the complementary action of the two agents on different tumor compartments [27], [33]. The main targets for taxanes are tumor cells and it is recognized that a significant issue limiting their antitumor activity is post-extravasation transport within tumor tissue [4], [60] and as such their activity is higher in well perfused areas. The rationale for their enhanced performance with VDAs is that VDAs shut down flow primarily in the fragile abnormal neovasculature of poorly perfused tumor centers, whereas taxanes preferentially act upon well perfused areas such as the tumor periphery. It has also been suggested that trapping of the taxanes within the tumor may play a supplementary role [38] though it is notable that combined effects can still be obtained when a VDA is administered first [56]. Beyond these two effects, there can be other factors involved such as their combined action on the same tumor compartment. With regards to the latter point it should be noted that taxanes, particularly at lower dose levels, can also induce antivascular effects [61].

Despite differences in the mechanisms of antivascular action between these two classes of VDAs, both involve a significant shutdown of tumor perfusion and both have interacted well with taxanes in terms of antitumor activity. The results of the present paper are consistent with these findings. The USMB treatments induced a rapid blood flow reduction, preferentially within the tumor centers. As the tumors were within the beamwidth of the transducer, it is interpreted that this differential spatial response, which also occurs with VDAs, is primarily due to differences in the nature of the vasculature at the center and periphery of the tumor. The combined therapy significantly inhibited tumor growth relative to individual treatments and produced tumor shrinkage. As is the case for VDAs, it is reasonable to postulate that a primary mode of action that is responsible for the combined effects is the complementary action of the two therapies. It is also possible that chemotherapeutic agent trapping may have contributed to the observed effects and in this regard it is notable that the docetaxel injection occurred 10 minutes prior to the commencement of USMB treatments. Indeed this delay time was selected based on previous results for DMXAA combined with the taxane paclitaxel, which showed increased antitumor activity when the VDA was administered subsequent to paclitaxel. While these two factors may be the dominant contributors to the combined effects observed here it is also possible that other considerations also play a role. Combined antivascular effects may be occurring for example, though it is notable that in the acute data we did not observe significant differences in flow inhibition between the combined treatment versus USMB-only groups. The process of USMB induced vascular damage is not well understood at present, though it has been established that USMBs can initiate an inflammatory response [62], [63], may be associated with platelet adhesion [64], and can cause ceramide pathway upregulation [65]. Further, as has been shown with VDAs and micelles [66], permeability enhancements can result in increased uptake as has also been shown with USMBs under conditions that produce vascular damage [53]. Clearly an improved understanding of the specific mechanisms of USMB antivascular effects, and their interaction with anticancer agents will be of interest in future work.

Therapeutic ultrasound is a rapidly emerging technique for the nonsurgical treatment of a range of solid tumor types [67]. The method employed in clinical work at present is to ablate tumor tissue with HIFU, which exploits the absorption of US energy by tissue. It is also under investigation as a means by which to promote the delivery of anticancer agents to tumor tissue [1], [3]. Hyperthermia is one method for accomplishing this, whereby mild temperature elevations induced by US can promote both the local release and uptake of therapeutic agents [68], [69]. US has also been employed to locally release drugs from novel encapsulated formulations [70], [71], in many cases through the induction of cavitation. The approach taken in this study was to employ a clinically approved class of drugs, which are also being investigated conjunction with VDAs in a number of clinical studies [38], [39]. An advantage of the approach described in the present study is that it does not involve the systemic injection of small molecule VDAs, which are associated with dose limiting side-effects [27]. A significant limitation of the USMB approach relative to systemically administered VDAs is that the therapy is localized and must be applied to a known tumor location that is situated in a region that is amenable to the delivery of controlled US exposures. Ablative therapeutic US can be applied to a range of tumor types and body locations such as liver, kidneys, brain, head and neck, pancreatic, prostate and breast. In the present study, prostate cancer was selected for evaluation since it is readily accessible to controlled therapeutic ultrasound exposures and docetaxel in combination with VDAs has previously been shown to be effective in achieving enhanced antitumor effects in prostate tumor cell lines [38]. Tumors located at these other sites are also suitable targets for the present approach. The antitumor effects of USMB treatments for these tumor types remains to be evaluated, and will presumably be influenced by their particular vascular properties, but it notable that a number of tumor cell lines have now been reported to be susceptible to vascular damage with USMBs, albeit not yet in combination with anticancer agents.

Ablative US involves the use of high US intensities and long treatment times (up to hours, depending on size) whereby a small focal volume is swept over a target volume. High intensity ultrasound, in the absence of injected MBs, has also been shown to be capable of damaging tumor microvasculature [72] and larger vessels [73]. The approach shown in the present study involves relatively low pressure levels compared to ablative US and can in principle be implemented on existing clinical therapeutic US systems. While in the present study the entire tumors were within the beam width, for larger clinically relevant tumors the focus will necessarily be swept using array and or mechanical scanning over the target volume, which will also enable for example differential exposure conditions at the tumor center and periphery. Further, the comparatively low pressure levels required will permit its implementation with larger transducer focal volumes, which could thereby reduce the time required to expose larger tumor volumes. It would also facilitate the exposure of tumors in more acoustically challenging locations, such as brain or liver where acoustic focusing due to ribs and tissue motion can be problematic for ablative therapy [74]. In moving towards clinical applications it will be important to ensure that acoustic exposure levels are controlled such that risks of potential damage to normal tissue present along the beam path before the target tumor tissue are minimized. As part of this, it will be necessary to clearly define what the damage thresholds are in the relevant non-target normal tissues.
